# Accelerating the domestication of a bioenergy crop: identifying and modelling morphological targets for sustainable yield increase in *Miscanthus*

**DOI:** 10.1093/jxb/ert225

**Published:** 2013-09-24

**Authors:** Paul Robson, Elaine Jensen, Sarah Hawkins, Simon R. White, Kim Kenobi, John Clifton-Brown, Iain Donnison, Kerrie Farrar

**Affiliations:** ^1^Institute of Biological, Environmental & Rural Sciences, Aberystwyth University, Gogerddan, Aberystwyth SY23 3EE, UK; ^2^MRC Biostatistics Unit, Institute of Public Health, University Forvie Site, Robinson Way, Cambridge CB2 0SR, UK; ^3^Centre for Plant Integrative Biology, Nottingham University, Nottingham LE12 5RD, UK

**Keywords:** bioenergy, biomass yield, domestication, *Miscanthus*, morphology, trait diversity.

## Abstract

To accelerate domestication of *Miscanthus*, an important energy crop, 244 replicated genotypes, including two different species and their hybrids, were analysed for morphological traits and biomass yield over three growing seasons following an establishment phase of 2 years in the largest *Miscanthus* diversity trial described to date. Stem and leaf traits were selected that contributed both directly and indirectly to total harvested biomass yield, and there was variation in all traits measured. Morphological diversity within the population was correlated with dry matter yield (DMY) both as individual traits and in combination, in order to determine the respective contributions of the traits to biomass accumulation and to identify breeding targets for yield improvement. Predictive morphometric analysis was possible at year 3 within *Miscanthus sinensis* genotypes but not between *M. sinensis*, *Miscanthus sacchariflorus*, and interspecific hybrids. Yield is a complex trait, and no single simple trait explained more than 33% of DMY, which varied from 1 to 5297g among genotypes within this trial. Associating simple traits increased the power of the morphological data to predict yield to 60%. Trait variety, in combination, enabled multiple ideotypes, thereby increasing the potential diversity of the crop for multiple growth locations and end uses. Both triploids and interspecific hybrids produced the highest mature yields, indicating that there is significant heterosis to be exploited within *Miscanthus* that might be overlooked in early selection screens within years 1–3. The potential for optimizing biomass yield by selecting on the basis of morphology is discussed.

## Introduction

In response to the challenges of maintaining energy security, mitigating climate change, and reducing the impact of peak oil in the face of increased demand, it is vital that sustainable bio-based energy and bulk chemicals are developed to substitute for petroleum-based products. Meeting this challenge will require the rapid improvement of entirely novel crops optimized for harvestable biomass. The targeted and accelerated domestication of dedicated energy crops over a small number of years represents an unprecedented challenge to plant breeding and requires the application of interdisciplinary approaches.

Although there are many potential sources of plant biomass, there is a specific need for dedicated biomass crops that perform well on suboptimal land, so as to minimize conflict with food production, and with low demand for energy-intensive inputs such as fertilizers. Harvested plant biomass primarily comprises fixed carbon, usually in the form of complex or simple polysaccharides and the energy-rich polymer lignin, ideally with little associated protein (Gomez *et al.*, 2008). The benefits of increasing harvestable yield in biomass crops include improved land use efficiency, economic viability, and the capture of more atmospheric carbon. However, increasing yield in biomass crops will largely target different traits to those modified during the domestication of many food crops, as domestication for food uses has focused largely on enhancing grain production, especially in cereals, at the expense of overall biomass accumulation (Sang, 2011). The use of high-energy food crops to produce bioenergy is inefficient when intensive annual agronomic practices are accounted for and reduces the availability of high-quality land for food production (Valentine *et al.*, 2012). To optimize biomass production, dedicated crops with efficient energy capture are required in which the photosynthate is optimized and partitioned predominantly to the harvestable vegetative structures, i.e. primarily to the stems.

In recent years, C4 grasses, in particular members of the perennial genus *Miscanthus*, have been identified as energy crops with global potential and are therefore excellent targets for improvement (reviewed recently by Brosse *et al.*, 2012, and van der Weijde *et al*., 2013). *Miscanthus* originates from diverse climates ranging from tropical Africa and South-East Asia up to Siberia, and is a highly productive temperate biomass crop due to its rapid biomass accumulation in temperate climates with low input requirements. Currently a single clone, *Miscanthus* × *giganteus* (*M. *× *giganteus*) is grown commercially. In order to diversify the crop and breed novel high-yielding varieties optimized for different environments and end uses, it is imperative to evaluate and utilize the wide genetic diversity present within the genus. Sixteen species of *Miscanthus* are currently described on GrassBase—The Online World Grass Flora (Clayton *et al.*, 2006 onwards). Among those with potential for development as biomass crops are *Miscanthus sinensis* and *Miscanthus sacchariflorus*, which each have very wide distributions including both tropical and temperate regions within Asia, and *Miscanthus floridulus*, which is limited to low altitudes and tropical areas (Clifton-Brown *et al.*, 2008*b*). *Miscanthus lutarioriparius*, a very tall type, is localized to one region of China and has previously been considered a subspecies of *M. sacchariflorus*. It is likely that breeding programmes will incorporate these diverse species to different degrees, both for suitability to the growing environment and in terms of biomass properties for end-use applications.

If *Miscanthus* is to make a significant contribution to providing sufficient biomass to fuel a low-carbon bioeconomy, it will need to be domesticated within the next two decades. Domestication is, in effect, the reduction in genetic variation within a population to increase the frequency of desired traits over undesired or neutral traits.

Unlike the domestication of the cereals, which was based purely on phenotypic (ideotype) selection, the domestication of *Miscanthus* will be genetically based, with genotypes conferring the desired traits in a range of environments being selected for recurrent selection (Johnson *et al.*, 1992). The majority of *Miscanthus* germplasm is either directly collected from the wild or is no more than one or two generations removed. In contrast to recent crop breeding, energy crops must be domesticated to retain and improve their innate resource use efficiency so that they are high yielding over successive growing seasons without the requirement for environmentally and economically costly fossil-fuel-based inputs. Since the widespread application of nitrogen fertilizers, crop breeding has focused almost entirely on selecting for yield increase with fertilizer rather than optimizing the plant’s inherent nitrogen-use efficiency. There is consequently an inherent dependence on fertilizer usage for the majority of the world’s food production, which is expensive both economically and environmentally. In domesticating a novel crop for biomass production, not only do we need to focus on different morphological traits, but we must also be mindful to ensure that our primary selections for biomass yield are not compromising other aspects of sustainable crop production in the future.

In terms of domestication then, *Miscanthus* breeders are seeking to reduce the frequency of alleles conferring undesirable traits in the breeding populations. Increasing the frequency of beneficial alleles and reducing the frequency of detrimental alleles is achieved through repeated cycles of recombination and selection. Typically, a breeding cycle takes up to 7 years (Casler, 2012); however, at least a few hundred generations may have been required for domestication in the past (Burger *et al.*, 2008). Increasing the selection pressure increases the rate of genetic gain (Moose & Mumm, 2008) but may also increase the concurrent loss of desirable variation in other traits.

Biomass accumulation is a complex process comprising multiple structural component traits. A key factor in breeding is to understand the correlations between traits and the extent to which they can be uncoupled. It is imperative that the interactions between traits, both positive and negative correlations, are well understood because selection for one trait can have profound effects on another (Farrar *et al.*, 2012).

A thorough understanding of the key traits that contribute to harvestable biomass yield in these grasses is required in order to drive genetic gain for accelerated domestication. High yield in terms of harvestable biomass per plant is a composite of multiple simple traits, which not only act in combination but also interact in complex ways, thereby enabling the selection of high-yielding plants via different trait-optimization strategies (i.e. through multiple ideotypes). The aims of this study were: (i) to determine the traits contributing to high biomass yields; (ii) to define high-yielding ideotypes for *Miscanthus*; and (iii) to identify genotypes for introduction into recurrent selection cycles for increased biomass production.

The ideotype should comprise the smallest possible number of simple traits that can be screened for maximum yield improvement. It was therefore essential to identify and dissect a range of composite morphological yield traits, and determine to what extent they can be optimized independently, or whether different traits are linked by physiological or genetic constraints. In order to determine the key traits comprising high yield in *Miscanthus* that may be used to predict high-yielding individuals, the phenotypic relationships were considered.

In 2004, a replicated trial of 244 genotypes, including *M. sinensis* and *M. sacchariflorus* species and a number of intra- and interspecific hybrids (including *M.*×*giganteus*), was planted in Aberystwyth to assess the diversity within genotypes previously brought to Europe, mainly by taxonomists and the horticultural industry. The genotypes were therefore pre-screened for survival in Europe and consequently limited to temperate types. The majority of the genotypes were diploid, but some tetraploid and triploid types were represented. Although the plants within this trial do not represent the full potential of *Miscanthus* for bioenergy applications, they provide an excellent resource in which to study diversity and to link plant morphology traits to yield in a temperate climate. The data described here represent mature plants during the third, fourth and fifth years of growth, as years 1 and 2 were considered to be of limited predictive value for long-term yield projections (Lewandowski *et al.*, 2000).

## Materials and methods

### Genetic resources

A total of 244 *Miscanthus* genotypes were assembled from smaller collections within Europe, including 102 genotypes from known collection points in China, Japa,n and South Korea, latitude range 32.3–43.6° N. Four clonal replicates were planted in a randomized trial as described previously (Clifton-Brown *et al.*, 2008*a*; Jensen *et al.*, 2011). The germplasm collection comprised 187 *M. sinensis* genotypes, 35 *M. sacchariflorus* genotypes, and 22 interspecific hybrid (henceforth referred to as hybrid) genotypes, including diploid, triploid, and tetraploid genotypes (Table 1).

**Table 1.** Frequency of species and ploidy in the trial genotypesThe replicated trial consisted of a total of 244 genotypes of different ploidy levels, comprising *M. sinensis, M. sacchariflorus*, and their hybrids.

	Diploid	Triploid	Tetraploid	Total
*M. sinensis*	184	3	0	187
*M. sacchariflorus*	18	1	16	35
Hybrid	10	8	4	22
Total	212	12	20	244

### Trial conditions

The trial was established on a sloping field (52° 26’ N 04° 01’ W) near Aberystwyth on the west coast of Wales, exposed to winds from the south and west. The soil is classified as a dystric cambisol and a dystric gleysol depending on spatial variation in drainage (FAO, 1988) with a stone fraction (particles >2mm) of approximately 50% of the soil mass in the 0–40cm layer.

Climate data (rainfall, temperature, and radiation) were obtained from the Gogerddan weather station (52° 25’ N 04° 01’ W). Average monthly rainfall for the years 2007, 2008, and 2009 was 109, 113, and 98%, respectively, of the long-term monthly average for Gogerddan (86.5cm). Monthly average maximum and minimum temperatures for 2007, 2008, and 2009 were similar to the long-term mean. The average annual temperature of 2007–2009 was 10.5 °C compared with the long-term average of 9.7 °C. Soil temperatures between 2007 and 2009 at a depth of 5cm did not fall below –1 °C. Solar radiation in 2007 and 2009 was higher than the long-term average of 9.4 MJ m^–2^ d^–1^ (104 and 105%, respectively), while 2008 was the same as the long-term average (Jensen *et al.*, 2011; Robson *et al.*, 2012).

### Phenotyping the structural components of *Miscanthus*

Following an establishment phase of two growing seasons, extensive phenotyping of mature plants was undertaken in year 3 (Y3) (2007), Y4 (2008), and Y5 (2009), which are considered to be the first ‘mature’ years under UK conditions (Clifton-Brown *et al.*, 2008*a*). A theoretical model describing the impact of trait variation on yield in *Miscanthus* guided trait selection. Stem traits were considered to be direct components of biomass, and other traits including leaf development (leaf length and width) and plant stature were hypothesized to affect light capture and water relations. Other key yield components—flowering time, emergence, and senescence—were also measured and have been described previously (Jensen *et al.*, 2011; Robson *et al*., 2012, 2013). Canopy height measurements were taken at fortnightly intervals throughout the growing season as the height at which the majority of light was intercepted by the canopy. At the end of the growing season (October), the following biomass component traits were measured: basal diameter (the diameter of the clump at ground level), transect count (the number of stems counted along a transect inserted through the middle of the clump at approximately half canopy height), stem diameter (the average of three stem diameters taken at mid-internode at approximately half canopy height), and tallest stem (the height to the highest part of the stem, excluding the flower, if present). Additional morphometric measurements were taken at least once during this time period: leaf length (length of the leaf blade from petiole to tip, excluding the leaf sheath), leaf width (width of the leaf blade at approximately half leaf length), and plant stature (stem angle and leaf angle).

### Dry matter yield (DMY)

The biomass yield of each mature plant was analysed in the February following the growing season. Harvest was delayed to spring to reflect current management practice. The spring harvest improves biomass quality in terms of moisture and nutrient content, despite a concurrent loss of biomass yield relative to the peak yield (Clifton-Brown *et al.*, 2007). Plants were harvested at a height of approximately 5cm from the soil surface and the whole above-ground biomass was passed through a silage chopper. The chopped plant material was collected in a plastic sack and weighed to determined total fresh weight (FW_total_). A subsample from the bulk sample of approximately 250g was removed, placed in a paper bag, and weighed to determine the subsample wet weight (FW_subsample_). The subsample was then dried to a constant weight at 60 °C and the percentage dry weight calculated (DW_subsample_). The percentage dry weight was used to calculate the total dry weight of the bulk sample: DW_total_=FW_total_×(DW_subsample_/FW_subsample_).

### Statistical analysis

The morphological traits were measured and considered as explanatory variables in several linear regression models for the total DMY. There were two intrinsic traits, species and ploidy, which were invariant for each plant. In addition, the year and replicate block were considered as two further traits, which partly captured any otherwise unmeasured environmental effects, specifically developmental and climatic differences between years, and spatial differences between replicates. The different linear models were used to compare the ability of individual explanatory variables and subsets of explanatory variables to predict DMY. To improve model fit and predictive ability, transformations of variables were considered. For all regression models, adjusted *r* ^2^ values are reported to indicate predictive ability. Furthermore, to aid comparison of nested models (i.e. variables included in one model are a subset of another), the Akaike information criterion (AIC) is reported as a measure of model fit in selected cases.

All tabulations, plots and, statistical analyses were performed in the GNU R statistical software (R Development Core Team, 2012). Linear regressions were performed using the lm command.

## Results

### *Trait diversity within* Miscanthus *species*

**Fig. 1.**
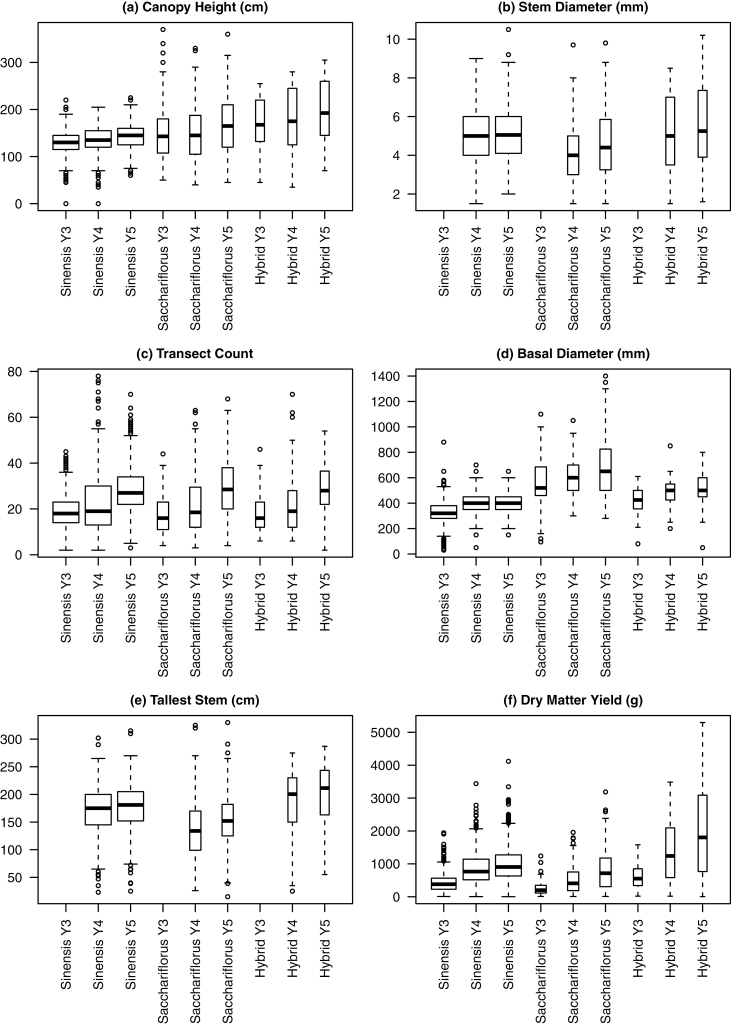
Summary of measured traits as boxplots for all plants by species (*M. sinensis*, *M. sacchariflorus*, and hybrid) and year (Y3, Y4, and Y5).

Overall morphological diversity within the trial was extremely high with tallest stem measurements ranging from 15 to 330cm, stem diameter from 1.5 to 10.5mm, leaf length from 12 to 100cm, leaf width from 0.2 to 3.6cm, and transect counts of 2 to 78. *M. sinensis* and *M. sacchariflorus* and their interspecific hybrids exhibited different morphologies. *M. sinensis* plants typically were shorter (canopy height <2 m) with a clumped base, while *M. sacchariflorus* plants could be taller with a spreading base. The hybrids were the tallest group on average, and had an intermediate base. Despite representing the largest group (*n*=187), the *M. sinensis* group show the smallest trait diversity in terms of canopy height and basal diameter (Fig. 1a, d).

### Effect of maturity on different species

**Fig. 2.**
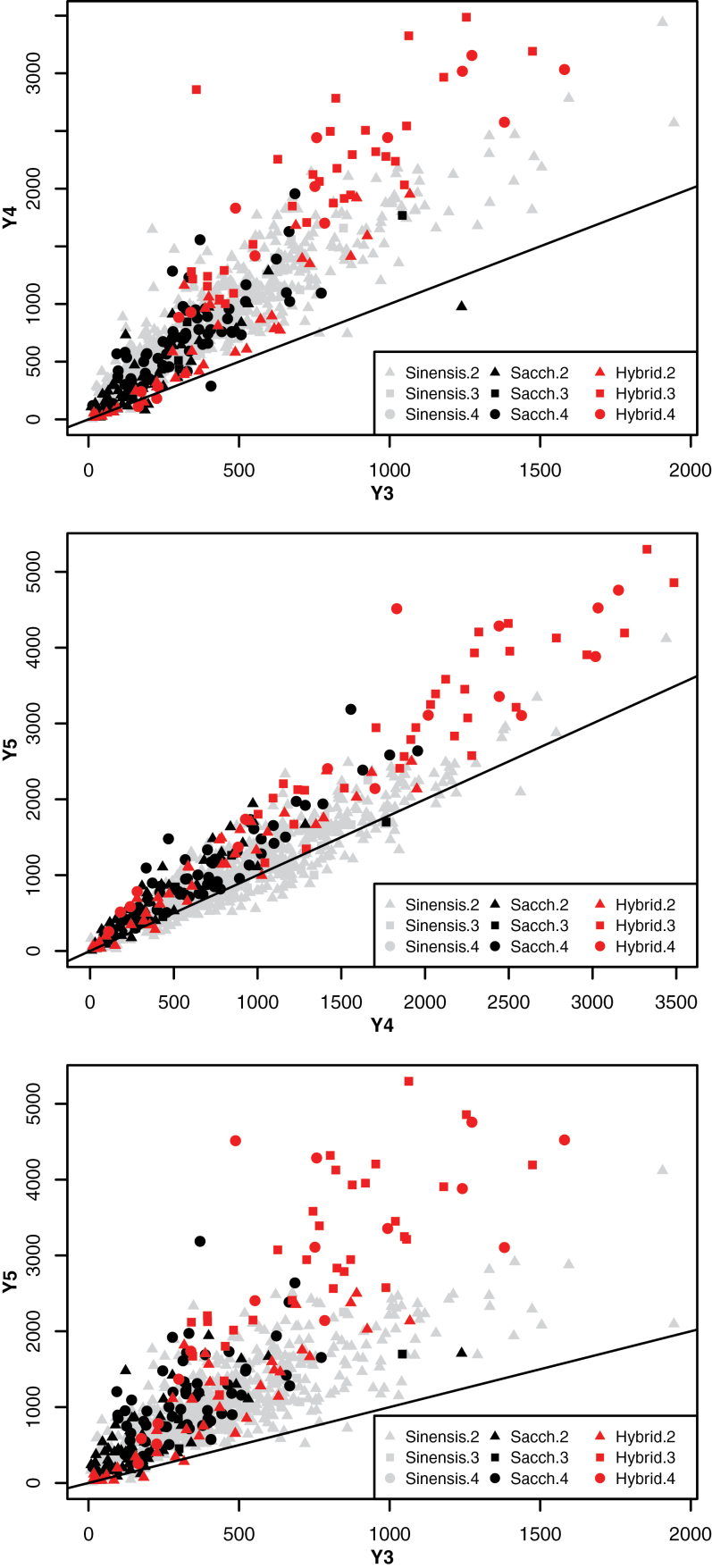
Year-on-year trends for DMY for all plants by species: *M. sinensis* (grey), *M. sacchariflorus* (black), and hybrid (blue); and by ploidy: diploid (triangle), triploid (square), and tetraploid (circle). The line of equality, *x*=*y*, is represented by a black line.

The first recorded harvest occurred following the third growing season (Y3); however, the DMY continued to increase over the 3 years of this study (Y3–5, Fig. 1f). DMY increased between Y3 and Y5 for all but 15 out of 976 plants, indicating that all genotypes used in this experiment had not achieved maximum yield potential at Y3. Plotting the DMY for each plant in Y3 against Y4, Y4 against Y5, and Y3 against Y5 demonstrated that *M. sinensis* plants had a lower DMY gain between Y4 and Y5 compared with *M. sacchariflorus* and hybrid genotypes (Fig. 2). This indicated that *M. sinensis* reached maximum yield faster, and that the yield of both *M. sacchariflorus* and hybrids continued to increase annually until at least Y5 under UK conditions.

### Effect of intrinsic and environmental traits on DMY

**Fig. 3.**
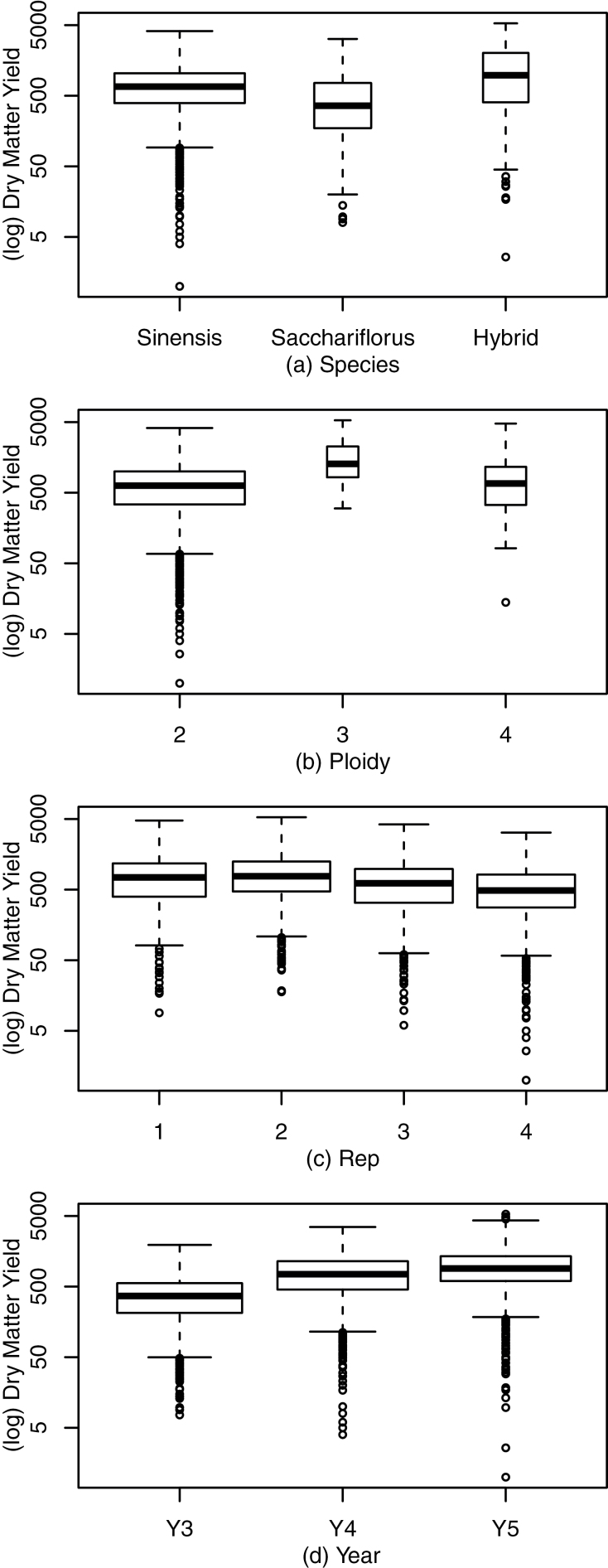
Comparison of log(DMY) across experimental traits of species (a), ploidy (b), and intrinsic traits of replicate block (c) and year (d). Widths of boxes indicate the number of plants within each group (see Table 1).

DMY varied from 1 to 5297g in the trial over the 3 years. DMY means for *M. sinensis*, *M. sacchariflorus*, and hybrids were calculated using all 244 genotypes in the trial. There were more *M. sinensis* genotypes in the trial, reflecting the relative availability of the different species in Europe at the time the experiment was established (Table 1; Clifton-Brown *et al.*, 2008*a*). Furthermore, there was a non-normal distribution of traits, including yield, within the trial. The effects of the intrinsic and environmental traits on the DMY were considered using the natural logarithm of the DMY due to the skew in distribution of DMY. The distribution of log(DMY) is shown as box and whisker plots in Fig. 3, subgrouped by species, ploidy, replicate block, and year; the width of each box corresponds to the size of each subgroup (see Table 1).

Hybrid plants exhibited a higher median yield than parental species (Fig. 3a) and triploid plants exhibited a higher median yield than either diploids or tetraploids (Fig. 3b). There was a small replicate effect, with replicate 4 yielding less on average than replicates 1, 2, and 3 (Fig. 3c). These observations were consistent with the geography of the trial site, as replicate 4 was at the top of a hill and experienced more wind and more rapid water drainage than the lower replicates. Perhaps most striking was the year-on-year increase in DMY (Fig. 3d).

### Developing an ideotype

#### Linear regression of biomass traits—phenotypic correlations

**Fig. 4.**
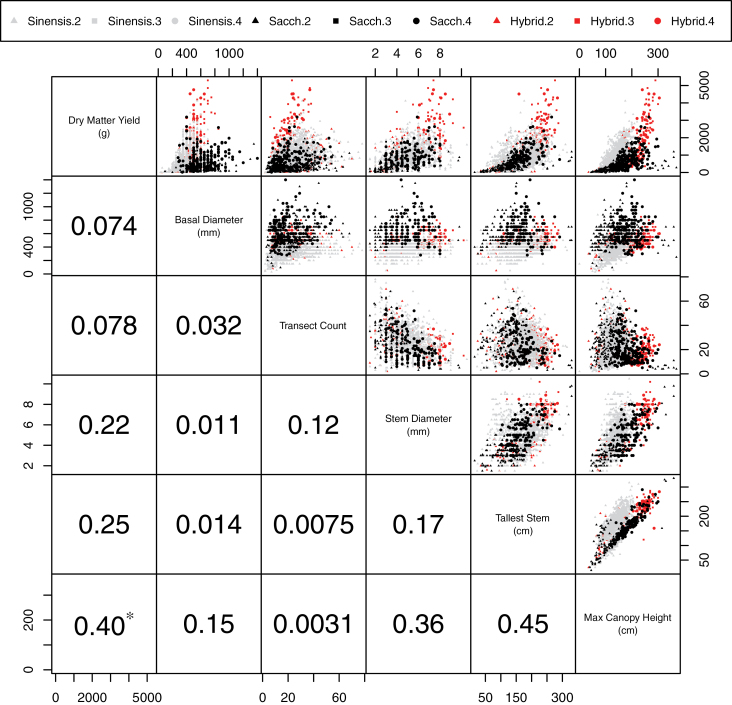
Pairwise trait plots for all replicates and associated *r* ^2^ values between traits for Y4 and Y5 by species: *M. sinensis* (grey), *M. sacchariflorus* (black), and hybrid (red); and by ploidy: diploid (triangle), triploid (square), and tetraploid (circle). The asterisk indicates the highest trait association with yield.

Pairwise plots between the untransformed measured traits (excluding leaf width and leaf length) and Pearson’s correlation *r* ^2^ coefficient between traits are shown in Fig. 4; no single trait predicted yield with any accuracy. The different species displayed different plant forms; *M. sinensis* had small basal diameters and high stem numbers (transect count), while *M. sacchariflorus* types had larger basal diameters and were otherwise somewhat diverse for morphological characters. The hybrid group was highly morphologically diverse, despite being small in number and containing the majority of high-yielding plants. There was an overall relationship between plant height (tallest stem/canopy height) and stem diameter, indicating that the former may be physically restricted by the latter. Conversely, there was a predominantly negative relationship between number of stems (transect count) and stem diameter. Basal diameter was not correlated with stem diameter, which would be expected if stem number were consistent among genotypes but is unsurprising given the high variation observed in the number of stems (transect count=2–78). Plant height (tallest stem/canopy height) and transect count appeared to be independent of one another, especially in the *M. sacchariflorus* group, although there were no plants within the trial with high numbers of tall stems.

**Fig. 5.**
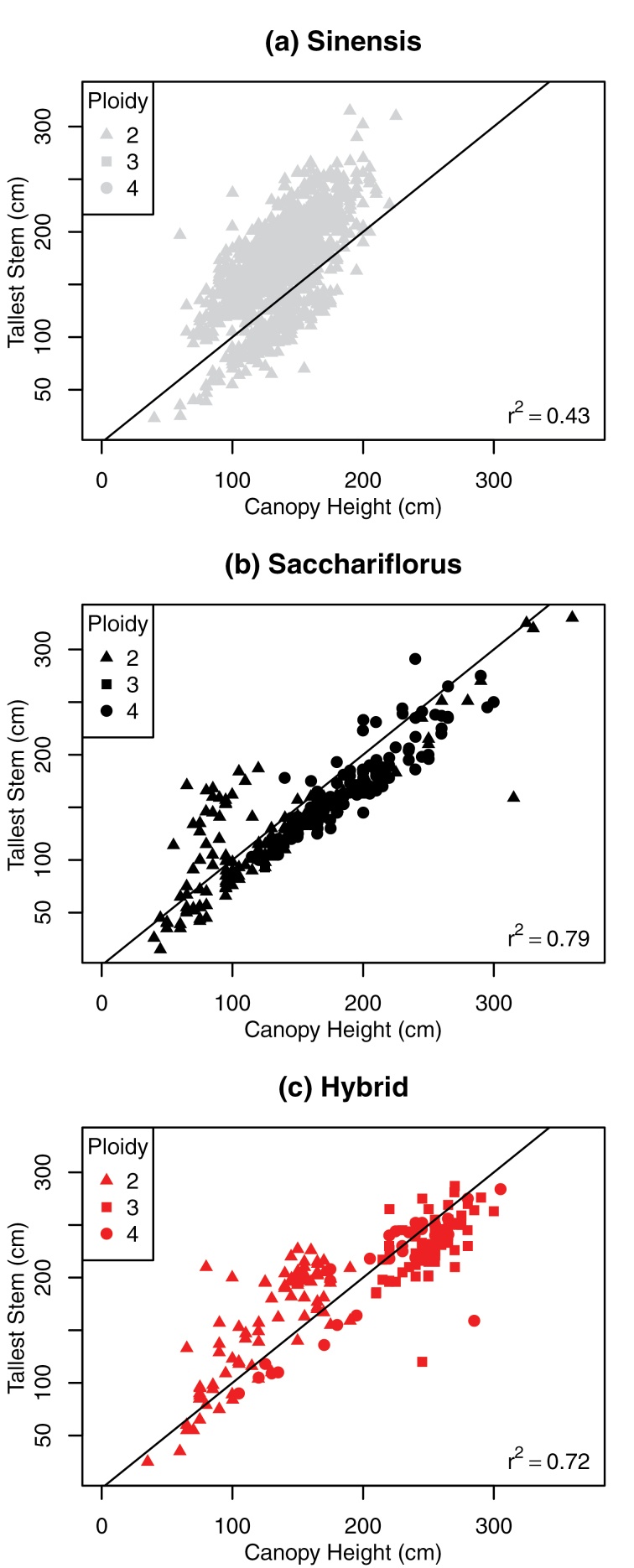
Pairwise trait plots between tallest stem and canopy height for all replicates for Y4 and Y5 by species: *M. sinensis* (grey), *M. sacchariflorus* (black), and hybrid (red); and by ploidy: diploid (triangle), triploid (square) and tetraploid (circle).

Canopy height had the highest correlation with yield, but this is a complex trait comprising stem and leaf height/length and angle traits. The simple trait with the highest correlation to yield was tallest stem, which was clearly a component of canopy height but was substantially less correlated with yield. Further analysis of the relationship between canopy height and tallest stem revealed that there were broadly two linear relationships between the two traits, which were represented differently within the three species groups (Fig. 5); this may represent the phenology of the plant as the majority of *M. sinensis* flowered in this trial while the majority of the other species did not.

**Fig. 6.**
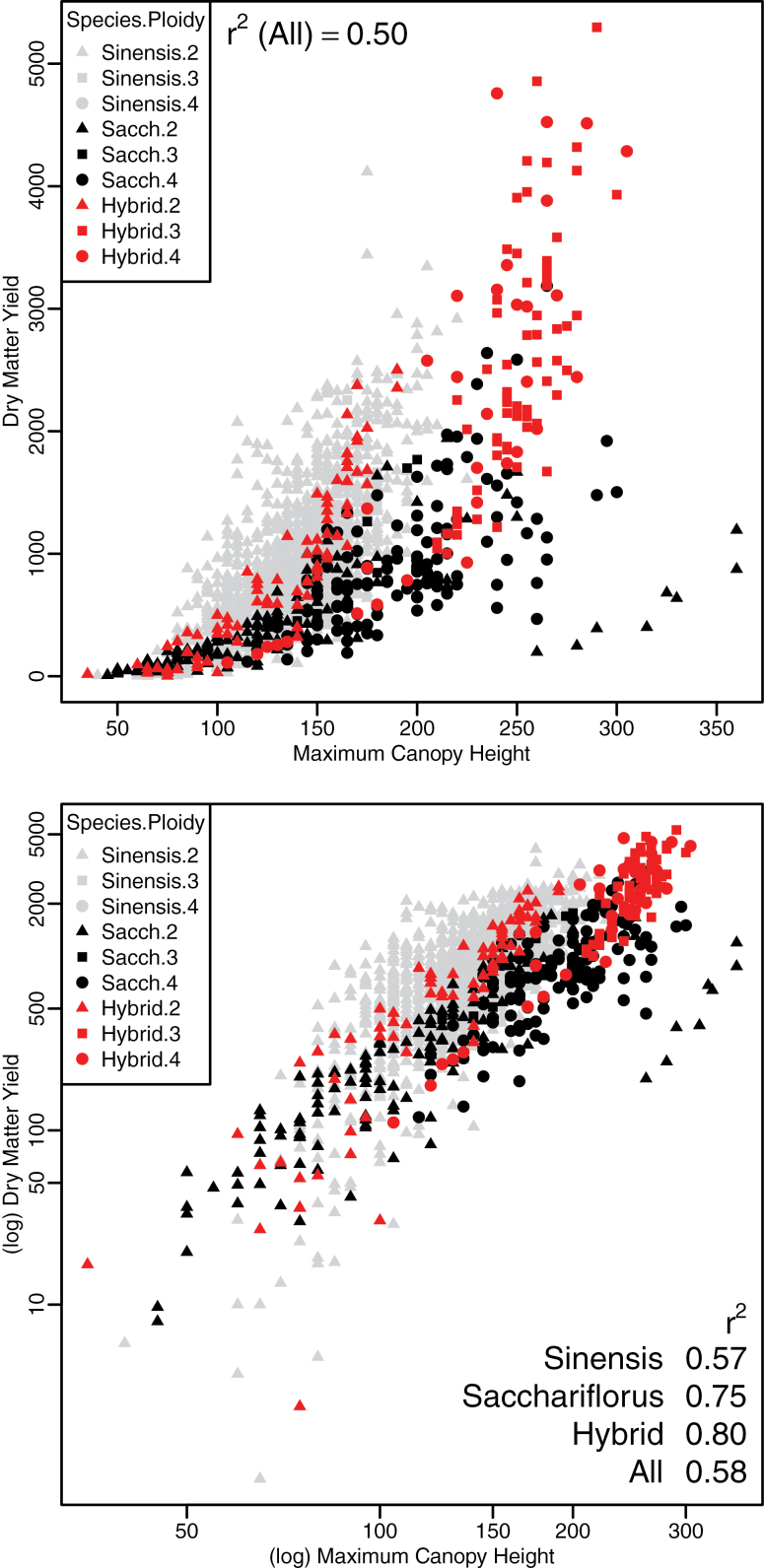
Pairwise trait plots between dry DMY and canopy height for all replicates for Y4 and Y5 for untransformed (upper plot) and log transformed (lower plot) data by species: *M. sinensis* (grey), *M. sacchariflorus* (black), and hybrid (red); and by ploidy: diploid (triangle), triploid (square), and tetraploid (circle).

Explanatory plots of the data demonstrated that the association between each trait and DMY was non-linear in many cases, including the relationship of DMY to maximum canopy height, especially for high-yielding plants (Fig. 6, upper plot). Using log transformation (Fig. 6, lower plot), the association was reasonably linear, although there was departure for smaller yields, which were of less value in terms of optimizing the model for high-yielding *Miscanthus*. Log transformations were used for all traits for subsequent linear modelling. Furthermore, the exploratory analysis (Figs 1–3) indicated that the majority of plants were not yet mature in Y3 for the majority of plants, so Y4 and Y5 data only were included in subsequent analysis.

The morphological diversity observed within the trial indicated that multiple simple traits may be optimized independently of one another to increase yield. The four simple traits measured in Y4 and Y5 were correlated with yield both individually and in combination to determine the combination of measurements that best predicted yield (Table 2). Although a combination of all four gave the highest prediction (*r* ^2^=0.601), removing basal diameter did not have a great impact (*r* ^2^=0.589). However, no other combination had the predictive power of canopy height alone: *r* ^2^ log(maximum canopy height) vs log(DMY)=0.55.

**Table 2.** Comparison of simple linear models for log(DMY)Comparison of models for log(DMY) (Y3 and Y4) using one-time measurement traits, i.e. excluding maximum canopy height as it is a function of multiple measurements. The adjusted *r* ^2^ values indicate the proportion of variability in DMY explained by the included traits (the first four rows correspond to first column entries in Table 3). The AIC compares models, accounting for how well they fit the observed data and how many traits are included; lower values indicate better-fitting models.

Models for log(DMY) using:					
log(basal diameter)	log(transect count)	log(tallest stem)	log(stem diameter)	Adjusted *r* ^2^	AIC
					–890.7
✓				0.078	–1042.0
	✓			0.068	–1021.9
		✓		0.339	–1670.2
			✓	0.227	–1376.2
✓	✓			0.448	–2007.9
✓		✓		0.442	–1987.0
	✓	✓		0.422	–1923.3
✓			✓	0.276	–1497.4
	✓		✓	0.443	–1990.7
		✓	✓	0.413	–1891.6
✓	✓	✓		0.448	–2007.9
✓	✓		✓	0.465	–2067.4
✓		✓	✓	0.442	–1987.0
	✓	✓	✓	0.589	–2563.6
✓	✓	✓	✓	**0.601**	**–2620.6**

#### Statistical yield modelling incorporating additional morphometric measurements

Independent linear models regressing log(DMY) against each explanatory variable in turn were performed. Of these, only log(tallest stem) and log(stem diameter) explained more than 10% of the observed variation in log(DMY), (adjusted *r* ^2^ >0.1; Table 3). The individual trait that explained the most variation was the logarithm of the maximum canopy height (adjusted *r* ^2^=0.55), but this is a complex trait comprising aspects of stem and leaf morphology. Tallest stem, as would be predicted due to the strong pairwise correlation with maximum canopy height, was the most predictive single simple trait (adjusted *r* ^2^=0.339; Table 3).

**Table 3.** Complex linear models for log(DMY)Linear models for log(DMY) (Y4 and Y5) showing the effect of each trait separately (unadjusted column) and then adjusting for the inclusion of all other traits.

Trait	Separate trait effects (unadjusted)				Linear model including all traits		
	Coefficient	SE	*P* value	Adjusted *r* ^2^	Coefficient	SE	*P* value
Intercept/base line					–6.753	0.366	<0.001
Sacchariflorus	–0.919	0.075	<0.001	0.0782	–0.685	0.058	<0.001
Sinensis	–0.420	0.062	<0.001	0.0782	0.005	0.05	0.926
Ploidy 3	0.943	0.080	<0.001	0.0699	0.269	0.059	<0.001
Ploidy 4	0.205	0.063	0.001	0.0699	0.336	0.056	<0.001
Leaf angle 0.5	0.203	0.064	0.002	0.0087	–0.031	0.053	0.563
Leaf angle 1	0.294	0.069	<0.001	0.0087	–0.095	0.058	0.101
Stem angle 2	0.311	0.038	<0.001	0.0364	0.066	0.028	0.018
Stem angle 3	0.320	0.066	<0.001	0.0364	0.068	0.043	0.112
Stem angle 4	–0.092	0.161	0.567	0.0364	–0.238	0.097	0.014
log(basal diameter)	0.754	0.060	<0.001	0.0776	0.627	0.049	<0.001
log(transect count)	0.411	0.035	<0.001	0.0677	0.604	0.024	<0.001
log(tallest stem)	1.450	0.048	<0.001	0.3390	0.958	0.041	<0.001
log(stem diameter)	1.220	0.052	<0.001	0.2270	0.731	0.048	<0.001
log(leaf width)	0.513	0.044	<0.001	0.0656	–0.016	0.038	0.680
log(leaf length)	0.610	0.054	<0.001	0.0628	0.448	0.040	<0.001
					Adjusted *r* ^2^=0.672		
					AIC=–2976.5		

A linear model incorporating all traits is presented in the second column of Table 3, which results in a very high adjusted *r* ^2^ of 0.672. Thus, without any considerations apart from log transformations, 67% of the variation in yield was explained. Thus, a complex model including intrinsic traits (genotype and ploidy) as well as non-intrinsic traits (basal diameter, transect count, stem diameter, tallest stem, leaf length and width, and leaf and stem angle) gave a prediction of <10% more than a simple model that consisted of three simple traits: transect count, tallest stem, and stem diameter.

## Discussion

Few studies have been conducted to date including diverse *Miscanthus* genotypes, as most research has focused on the commercial clone *M. *× *giganteus*. These studies have been reviewed recently (Anderson *et al.*, 2011; van der Weijde *et al*., 2013) and demonstrate both the potential for growing *Miscanthus* over a very wide geographical area, across Asia, Europe and the USA, and also the major effect of climate on yield. Most trials including **M. *× *giganteus** have been conducted in Europe where harvested yields have been reported as 10–30 t DW ha^–1^; the highest yields (up to 44 t DW ha ^–1^ at the end of the growing season post-senescence) were reported from a trial in Illinois (Heaton *et al.*, 2008). Comparisons between *Miscanthus* and other species such as bamboo (Hong *et al.*, 2011), giant reed (*Arundo donax*; Angelini *et al.*, 2009) and other C4 grasses (reviewed by van der Weijde *et al*., 2013) consistently show *Miscanthus* to be among the most productive plant species for biomass production. In practice. a mixture of crops will be developed for use in different locations and for different end uses, and *Miscanthus* is likely to be a key constituent of the mix.

The main factor limiting the deployment of *Miscanthus* to date is the lack of domesticated material of sufficiently predictable high yield, despite a wealth of natural diversity available. Morphological evaluation is a relatively rapid method for screening diverse material to be entered into restricted breeding cycles for rapid domestication and crop improvement.

### Defining the ideotype

#### Simple correlations

For a given plant, there were mechanistic and biological relationships between several of the measured traits, most obviously between maximum canopy height and tallest stem. Maximum canopy height was the single measured trait that best described yield (*r* ^2^=0.4, increasing to *r* ^2^=0.55 for log values), and this correlation was stronger for *M. sacchariflorus* and the hybrid populations independently (Fig. 6, lower panel). Canopy height is a complex trait comprising stem height, leaf length, and stem and leaf angle, so, while being relatively easy to measure in the field, it is likely to require further dissection to identify individual genes regulating the components of this trait for targeted selection. Tallest stem has the highest correlation of the simple traits with yield (*r* ^2^=0.25; Fig. 4), which might be expected given that stem is a component of canopy height. While tallest stem was highly correlated with canopy height (*r* ^2^=0.45; Fig. 4), further analysis revealed that there were in fact at least two different relationships between the traits, which may represent the phenology of the plant (Fig. 5). The most likely explanation is that there is a linear relationship between canopy height and tallest stem for vegetative growth, which is perturbed by the extension of the panicle above the canopy; for example, Zub *et al.* (2011) reported an average of 30cm between canopy and panicle height. The majority of *M. sacchariflorus* types do no flower under UK conditions and demonstrate a simple 1:1 relationship between tallest stem and canopy height (Fig. 5b), while the second, less-well-defined relationship was observed for the majority of *M. sinensis* types that did flower in this trial (Fig. 5a). These data indicate that the mass of flowering stem above the vegetative canopy does not constitute a significant amount of biomass yield and that a new measurement ‘tallest stem to uppermost true leaf’ may be a valid simple trait to measure in future.

The two most informative traits following canopy height/tallest stem were stem diameter and transect count. While it was not surprising that a greater stem diameter was indicative of higher biomass yield, it was also confounded by the mechanics of stem growth, as a correlation existed between stem height and stem diameter (*r* ^2^=0.17). Interestingly, the correlation was higher for canopy height and stem diameter (*r* ^2^=0.36; Fig. 4), i.e. the additional flower stalk did not require a substantial stem diameter to support it, again indicating that the flowering stem did not contribute much in the way of biomass.

#### Complex correlations

Taking the simple traits both individually and in combination, it was possible to construct a simple model to predict optimization strategies for increasing yield. With the potential exception of transect count and stem diameter, the majority of traits did not appear to be negatively correlated, indicating that each could be optimized without unintended yield loss. Transect count, tallest stem, and stem diameter measurements predicted approximately 60% of yield, which increased to <70% with the addition of a further seven traits. Thus, these three measurements can account for the majority of the heritable yield, with environmental factors, or unmeasured traits, accounting for approximately one-third of the variation. The predictive value of the three traits may increase slightly if the tallest stem measurement were substituted with a new measurement, ‘tallest stem to uppermost true leaf’, to avoid the confounding effect of the flowering stem. Any pair of these traits, with the exception of basal diameter and stem diameter, gave an adjusted *r* ^2^ of 0.41–0.45.

A confounding aspect of morphological selection for yield is the effect of environmental variation on these traits. In a trial of 93 wild-collected *Miscanthus* populations comprising *M. sinensis*, *M. saccharflorus*, and *M. lutarioriparius*, Yan *et al.* (2012) demonstrated site×population interactions for most *M. sinensis* and *M. saccharflorus* traits. This emphasizes the importance of comparing data from trials at different locations. Comparisons of trait and yield data from different trials may not be simple due to different phenotyping methodology and yield measurements; for example, other studies have taken biomass measurements from a number of stems either at peak biomass or at the end of the growing season. In this study, we analysed whole plant biomass at the commercial time of harvest following winter. The diverse morphologies of the material within this trial are, however, well suited to provide robust relationships between constituent traits and yield.

The basic ideotype for high-yielding plants can be considered to comprise tall plants (above 1.5 m) with thick stems (at least 5mm) and, theoretically, high stem numbers. No plants in the trial used in this study exhibited both very tall and very numerous stems, but it should be possible to select for such plants, or attempt to generate them by crossing tall and highly tillering individuals and analysing the progeny. However, it may be that there is an optimal range for stem number, above which light interception to the canopy cannot be increased. Adding additional traits such as leaf length and width, and stem and leaf angle increased the yield prediction to 67% (Table 3). Given the diversity within the traits, it should be possible to generate high-yielding plants with different combinations of stem height, number, and diameter, for example *M. sinensis* plants tend to be shorter than *M. sacchariflorus* and hybrids, and so are likely to require higher tiller numbers to achieve high yields. Thus, there is considerable scope for diversity within the crop and hence targeted breeding for alternative end uses such as power generation or liquid fuel production.

### Potential for early morphometric prediction

As for other perennial plants, the possibility of a juvenile phase in *Miscanthus* severely hinders early phenotypic selection of mature traits. Years 1 and 2 were not measured in this experiment, as they have previously been considered ‘immature’ for *Miscanthus* in the UK, and were considered to constitute an establishment phase during which time there was little economic value to harvesting the crop. However, in this trial, the yield in Y3 was significantly lower than for Y4 and Y5, reflecting the fact that the plants had not all reached their full yield potential by Y3 in Aberystwyth. The data shown in Fig. 1 indicate that there are strong differences between *M. sacchariflorus*, *M. sinensis*, and hybrid groups, and that, while *M. sinensis* types may be approaching maturity at Y3, for both *M. sacchariflorus* and interspecific hybrids this may not occur until at least Y4, i.e. *M. sinensis* types may reach their yield potential at least a year earlier than *M. sacchariflorus* and the hybrids. Furthermore, while it may be possible to select high-yielding genotypes *within* species at Y3, it is not possible to predict the highest yielding genotypes within a mixed population.

The maturation phase in *Miscanthus* appears to consist firstly of individual ramets reaching a certain phenotype, in terms of height and diameter, while yield increases in subsequent years are primarily due to the production of increased numbers of ramets, as captured by the transect count measurement (Fig. 1). In a smaller study of 20 clones, Zub *et al.* (2011) identified plant height and shoot diameter as the morphological traits best correlated with yield in Y2 and Y3. This has important implications in terms of selecting genes for targeted improvement in a molecular breeding approach, as it may be these ‘mature’ traits that have the greatest potential in terms of long-term yield increase once the obvious increases in canopy height have been made.

### Genetic gain

In domesticating a new crop, one of the greatest challenges is to reduce the genetic complexity and select only those alleles conferring desirable traits. In order to make rapid gains, stringent criteria must be applied to eradicate excess allelic diversity from the breeding pool: the more stringent the selection, the more rapid the rate of improvement (Moose & Mumm, 2008). The primary traits defining biomass yields are those intrinsic to yield, i.e. stem height, diameter, and number, and these three traits alone predicted 59% of yield. However, the addition of traits such as leaf length and width, and stem and leaf angle increased the yield prediction to 67%. It is likely that, once the variation within the intrinsic traits is sufficiently reduced (to taller, thicker, more numerous stems), these other traits will play a greater role in yield optimization, for example through optimizing light capture by the canopy. In practice, the reduction in complexity (primarily via morphological selection) has led to genetic bottlenecking in the majority of modern crops, which is problematic for continued improvement. Historically, selection was based predominantly on traits associated with yield gains and agronomic practice, thereby inadvertently reducing natural variation for other important traits such as stress resistance (Doebley *et al.*, 2006). While yield increase is the primary aim, it is important to ensure that diversity for other desirable traits such as nitrogen-use efficiency, abiotic stress tolerance, composition, and longevity is retained for subsequent selection, so ideally one would select very few plants with multiple good traits.

Phenotypic and genetic correlations in plants are poorly understood (Waitt & Levin, 1998) and are complicated by the low heritability of key traits determining yield. Perennial plants demonstrate high levels of developmental plasticity, enabling them to survive throughout the year and over multiple growing seasons. Although this trial was conducted in a single location, significant differences were observed across the replicates, indicating that environmental variation was not eliminated in this experiment.

### *Implications for domestication/breeding of *Miscanthus *as a novel crop for biomass production*

The domestication of *Miscanthus* will differ from historic crop domestications in a number of ways. Not only are different traits being selected for, but following collection and characterization of wild material, promising genotypes from extremely diverse origins can be crossed whereas only closely located neighbours would have come into contact in early domestication events. Additionally, genetic gains may be made rapidly by isolation of diverse individuals targeted for recurrent selection without genetic drag from unselected genotypes as would have happened in the past.

Direct selection of desirable alleles is confounded by both genetic linkage, which requires high levels of recombination to uncouple genetically linked traits, and the complex nature of yield traits. Not only are the majority of yield-associated traits highly polygenic, but they are also phenotypically responsive to environmental conditions (Fig. 3c). In order to accelerate the domestication of robust high-yielding *Miscanthus* for growth over a wide range of geographies, trials are essential to determine which genotypes are consistently high yielding, and which are adapted to certain locations and climates. A suite of traits is desirable in an energy crop, including high establishment rate, cold tolerance, water-use efficiency, nitrogen-use efficiency, and optimized phenology. A direct way to select genotypes with a range of desirable traits is to gather diverse germplasm and screen for consistent high yield in diverse environments under the intended growing conditions, i.e. in plots on marginal land without addition of nitrogen fertilizer. As this is resource intensive, a morphological pre-screen is of great value in selecting high-yielding genotypes for multi-location testing. The selection of relatively few robust morphological traits that predict high yield is therefore of value for accelerating the selection of parents and progeny within the crossing cycle.

In addition to the variation observed between the parental species and their hybrids, there were also differences between plants of different ploidy levels, with triploid and tetraploid hybrids producing the highest yields in this trial. This raises the possibility that additional yield gains may be accumulated through exploitation of heterosis in *Miscanthus*. One option is to optimize yield within isolated groups within species and then intercross between groups to exploit heterosis, as is employed in maize breeding. This approach has been highly successful in switchgrass, with observations of 30–35% F1 heterosis superiority with respect to the best parent, even without selection for specific recombining ability (Casler, 2012).

The final critical difference between historic and modern domestication/breeding is the application of molecular markers. There is much discussion about how next-generation technologies can assist breeding (Moose & Mumm, 2008; Flavell, 2010, Casler, 2012). Methods range from simple selection of desirable candidate gene alleles to genome-wide selection of high-density molecular markers associated with desirable traits. In either case, existing models are based on breeding crops in which genetic diversity is much reduced in relation to their wild progenitors, and good phenotype–genotype associations are required. In focusing on simple traits in this study, we intend to enable targeted identification of genes regulating these traits through association studies and comparative genomics, in particular through exploitation of the synteny between *Miscanthus* and *Sorghum* genomes (Rooney *et al.*, 2007; Ma *et al.*, 2012; Olson *et al.*, 2012). In the long term, molecular selection of one kind or another will play a vital role in *Miscanthus* breeding; however, the short-term gains to be made via phenotypic selection should not be overlooked. The application of morphometric selection of *Miscanthus* for high yield simultaneously identifies genotypes for introduction into accelerated selection cycles and paves the way for phenotype–genotype studies, which will further accelerate the domestication and optimization of this promising crop and its relatives.

## Abbreviations:

AIC: Akaike information criterion
DMY: dry matter yield
DW: dry weight
FW: fresh weight
Y: year.
